# Dietary Lipids in Health and Disease

**DOI:** 10.1155/2019/5729498

**Published:** 2019-07-01

**Authors:** Kamal A. Amin, Abdelgadir M. Homeida, Reda H. El Mazoudy, Khalid S. Hashim, Mahdi Garelnabi

**Affiliations:** ^1^Department of Chemistry, Biochemistry, College of Science, Imam Abdulrahman Bin Faisal University, Dammam 31441, Saudi Arabia; ^2^Department of Biology, College of Science, Imam Abdulrahman Bin Faisal University, Dammam 31441, Saudi Arabia; ^3^Department of Biochemistry, Beni Suef University, Beni Suef, Egypt; ^4^Department of Biomedical and Nutritional Sciences, University of Massachusetts, Lowell, MA, USA

Lipids are important cellular and extracellular molecules. They are critical for cell structure, function, and energy, as well as organs and body insulation and protection. In addition, lipids metabolites are extremely essential for a wide range of cellular communication and metabolism. However, defective lipids metabolism is well known to modulate a wide range of chronic diseases such as cardiovascular diseases and cancer and several other genetically defective lipids pathways with severe health implications.

Major lipids in human health and diseases may broadly be classified as saturated and unsaturated fatty acids, sterols, phospholipids, sphingosine derivatives, and various other lipids metabolites such as eicosanoids

The Genome Wide Association Studies (GWAS) helped identify several genetically defective lipids pathways and linked them to causes of morbidity and mortality around the globe. This GWAS emerging technology has unraveled many metabolic defects associated with dietary lipids and causes of many health conditions such as obesity, cardiovascular neurodegenerative defects, and cancer. GWAS studies have demonstrated that many of the lipids disorders mechanisms are associated primarily with oxidative stress and inflammation. It is unclear how the environmental modulators and lifestyle are linked to these disorders, which prompt further investigations to determine the initial causes and possible intervention approaches.

This special issue covers some original research articles and reviews that seek to provide insight into the role of lipids in health and disease highlighting some critical links to lipoprotein metabolism and atherosclerosis.

The review by H. Takeuchi and M. Sugano described the complex linkages of industrial TFAs to cardiovascular which concluded that the relationship between dietary industrial TFAs and concentration of plasma cholesterol should be evaluated from the viewpoint of dietary patterns rather than TFAs alone. On the other hand, E. Derbyshire has demonstrated the role of omega-3/6 fatty acids in the treatment and management of ADHD suggesting that omega-3/6 fatty acids offer great promise as a suitable therapy for this childhood condition. However, F. Drobnic et al. assessed the omega-3 index response, in RBC, to supplemental EPA + DHA intake in the form of high purity and stable composition gums in elite summer athletes and concluded that supplementation of omega-3 FA helps improve the content of EPA + DHA in the RBC at 4 months in a dose-dependent manner.

M. Garelnabi et al. determined the effects of longer-term supplementation of mouse oxidized linoleic acid (OxLA) on plasma triglycerides on normal C57BL/6 mice. The study reported a 39% decrease in hepatic PPAR-*α* and a significant decrease in the plasma HDL levels compared to the mice that were fed diets of plain and linoleic acid supplemented chow, suggesting that the longer-term consumption of oxidized linoleic acid may predispose to atheropathogenesis. The other interesting study was authored by R. Ariyanti and B. Besral on the link between dyslipidemia associated with hypertension and incidence of CHD in Harapan Kita Hospital, National Cardiovascular Center, Jakarta. The study has reported that, after controlling for age, in hypertensive respondents, those with dyslipidemia were 18.1 times more likely to develop CHD compared with those without dyslipidemia, whereas in nonhypertensive subjects, those with dyslipidemia were 2.5 times more likely to develop CHD compared with those without dyslipidemia.

Certainly, these interesting articles point towards the need for additional studies to elaborate on the role of dietary lipids on health and diseases. We are confident this special issue will enrich our current understanding and further interest in lipids in health and disease.

