# Corrigendum to “Dietary Lipids in Health and Disease”

**DOI:** 10.1155/2020/5704752

**Published:** 2020-01-20

**Authors:** Kamal A. Amin, Abdelgadir M. Homeida, Reda H. ElMazoudy, Khalid S. Hashim, Mahdi Garelnabi

**Affiliations:** ^1^Department of Chemistry, Biochemistry, College of Science, Imam Abdulrahman Bin Faisal University, Dammam 31441, Saudi Arabia; ^2^Department of Biology, College of Science, Imam Abdulrahman Bin Faisal University, Dammam 31441, Saudi Arabia; ^3^Department of Biochemistry, Beni Suef University, Beni Suef, Egypt; ^4^Department of Biomedical and Nutritional Sciences, University of Massachusetts, Lowell, MA, USA

In the article titled “Dietary Lipids in Health and Disease” [1], there was an error in the third author's name format. The correct authors list and affiliations are shown above.
